# The Burden of Burnout among Healthcare Professionals of Intensive Care Units and Emergency Departments during the COVID-19 Pandemic: A Systematic Review

**DOI:** 10.3390/ijerph18158172

**Published:** 2021-08-02

**Authors:** Maria Rosaria Gualano, Tiziana Sinigaglia, Giuseppina Lo Moro, Stefano Rousset, Agnese Cremona, Fabrizio Bert, Roberta Siliquini

**Affiliations:** Department of Public Health Sciences, University of Turin, 10124 Turin, Italy; mariarosaria.gualano@unito.it (M.R.G.); tiziana.sinigaglia@unito.it (T.S.); stefano.rousset@unito.it (S.R.); agnese.cremona@unito.it (A.C.); fabrizio.bert@unito.it (F.B.); roberta.siliquini@unito.it (R.S.)

**Keywords:** burnout, intensive care unit, emergency department, COVID-19

## Abstract

The primary aim was to evaluate the burnout prevalence among healthcare workers (HCWs) in intensive care units (ICUs) and emergency departments (EDs) during the COVID-19 pandemic. The secondary aim was to identify factors associated with burnout in this population. A systematic review was conducted following PRISMA guidelines by searching PubMed, Embase, PsychINFO, and Scopus from 1 January to 24 November 2020. Studies with information about burnout prevalence/level during the pandemic regarding ICU/ED HCWs were eligible. A total of 927 records were identified. The selection resulted in 11 studies. Most studies were conducted in April/May 2020. Samples ranged from 15 to 12,596 participants. The prevalence of overall burnout ranged from 49.3% to 58%. Nurses seemed to be at higher risk. Both socio-demographic and work-related features were associated with burnout. Many pandemic-related variables were associated with burnout, e.g., shortage in resources, worry regarding COVID-19, and stigma. This review highlighted a substantial burnout prevalence among ICU/ED HCWs. However, this population has presented a high burnout prevalence for a long time, and there is not sufficient evidence to understand if such prevalence is currently increased. It also outlined modifiable factors and the need to improve emergency preparedness both from an individual and structural level.

## 1. Introduction

Since the initial outbreak of SARS-CoV-2 virus in China at the end of 2019, the COVID-19 epidemic has spread out rapidly all over the world [1] and, on 11 March 2020, the World Health Organization declared a state of global pandemic [2]. The rapid onset of this severe and challenging emergency has immediately put healthcare systems under enormous pressure, from both an organizational and clinical point of view. Management and organizational problems varied significantly among countries, based on the strengths and limitations of each specific national health system. However, the clinical challenge of treating a huge number of patients affected by an unknown infection, with scarce knowledge and limited resources, represented a significant and stressful working experience for healthcare workers (HCWs) around the world, especially for those at close contact with COVID-19 patients. Increased workload, little rest, feeling of inadequacy, fear of being infected or infecting others are all factors potentially associated with mental problems in HCWs facing the pandemic on the front line [3].

Several studies have been conducted in order to investigate the physical and mental consequences of the COVID-19 pandemic among physicians and nurses. High levels of stress, anxiety and depression were found in HCWs working in different European countries, such as Italy [4,5,6], Spain [7] and Germany [8]. Outside Europe, similar data have been found in Mexico [9], Singapore [10] and China [11,12,13]. These results have also been confirmed by systematic reviews, showing the dramatic and deleterious effects that the COVID-19 pandemic has exerted on the professionals involved in this unprecedented struggle [14,15,16]. However, another Chinese study unexpectedly showed a lower frequency of burnout in physicians and nurses working on the front line compared with those working in their usual wards [17]. Hence, despite the strong evidence mentioned above, data could still be potentially contradictory and more evidence is therefore required.

In addition, most of these data were obtained from studies conducted among HCWs in general. However, physicians and other professionals working in emergency departments and intensive care units are the ones dealing with the most critical patients and thus, are the most exposed to a high risk of contagion and work-related stress. In particular, anesthesiology is recognized as one of the most stressful medical specialties because of the intense workload and many responsibilities [18,19]. Hence, facing the COVID-19 pandemic on the front line could have represented an additional source of stress for HCWs, significantly increasing the risk of developing burnout syndrome. 

To our knowledge, no systematic reviews have comprehensively evaluated the impact of burnout syndrome among HCWs working in critical-care settings during the SARS-CoV2 outbreak. 

Therefore, the primary aim of this study was to evaluate the prevalence of burnout among HCWs working in intensive care units (ICUs) and emergency departments (EDs) during the COVID-19 pandemic. The secondary aim was to identify the potential factors associated with burnout in order to hypothesize strategies to prevent or reduce this heavy psychological burden in the most exposed HCW in times of emergency. 

## 2. Materials and Methods

### 2.1. Literature Search and Inclusion Criteria

Our search strategy included both free terms and specific thesaurus terms contained in the title or abstract related to burnout, ICU/ED HCWs and the COVID-19 pandemic. A complete scheme of the search strategy is reported in Supplementary Material S1. 

We performed a systematic search in PubMed, Embase, PsychINFO, and Scopus from 1 January 2020 to 24 November 2020. The Preferred Reporting Items for Systematic Reviews and Meta-Analyses (PRISMA) checklist was followed [20]. Inclusion criteria were: original studies giving information about the prevalence or level of burnout regarding HCW (including doctors, nurses, respiratory therapists, pharmacists, administrators) working in ICUs or EDs during COVID pandemic. Selection was restricted to studies published in English and those that were peer-reviewed. We excluded reviews and studies regarding students. Table 1 shows the PICOS strategy used for this review. Duplicates were removed. 

### 2.2. Study Selection, Data Extraction and Quality Assessment

First, papers were screened for title and abstract by the authors (SR, TS, GLM, AC) using Rayyan software [21]. Disagreements were solved by discussion. Then, the authors independently applied the inclusion and exclusion criteria to the full texts.

After the full-text screening, relevant information regarding articles, such as study type, country, population considered, sample size, and results, were extracted in a spreadsheet (SR, TS, GLM, AC). Reasons for exclusion were documented.

The methodological quality of the studies was assessed with the appraisal tool for cross-sectional studies (AXIS) [22]. Two authors (SR, TS) independently evaluated each study, and disagreements were solved by a third author (GLM). For each item of the assessment tool, a score of 1 was assigned if the study satisfied it in terms of methodological adequacy, otherwise the assigned score was 0. The percentage of items with a score of 1 is reported for each paper, providing an estimate of the overall methodological quality of the included study.

## 3. Results

**Figure 1:**
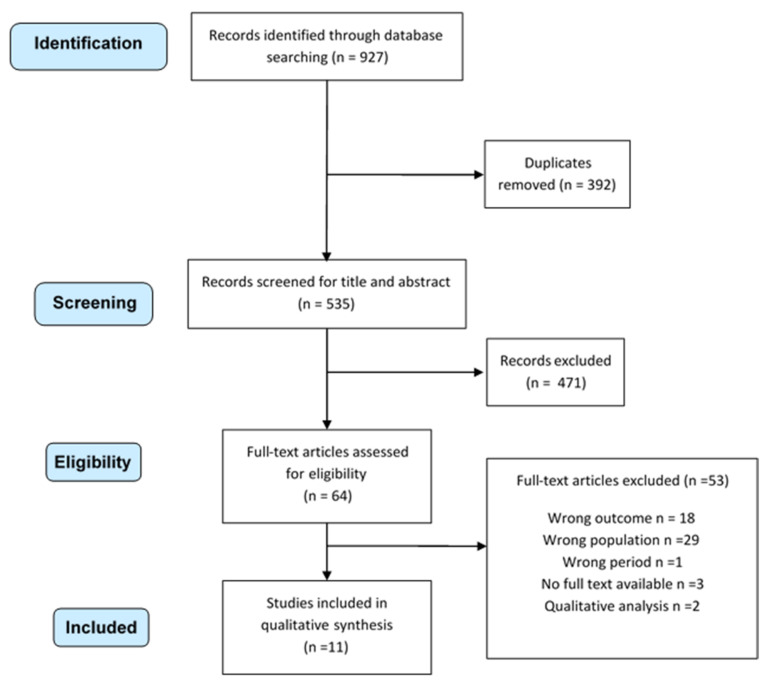
Systematic review selection process.

A total of 927 articles potentially useful to investigate burnout in the selected population were found: 236 in PubMed, 236 in Scopus, 444 in Embase, and 11 in PsychINFO. After excluding 392 duplicates, 536 articles were obtained and evaluated by title and abstract: 471 records were ruled out, and 64 were left to review completely by full text. After reading the full texts of all articles, 11 records were selected and enrolled for systematic review (Figure 1).

Table 2 shows the characteristics of the included papers. Two studies were conducted worldwide [23,24], while the rest of the surveys were conducted across seven different single countries (USA, Spain, Canada, Italy, Malaysia, China, Singapore) [25,26,27,28,29,30,31,32,33]. Four works had a declared funding source [26,27,32,33], and two works stated potential conflicts of interest [25,27].

The vast majority of the included studies were cross-sectional (N = 9) [25,26,27,28,29,30,31,32,33]. One study was a mixed method study [27], and one was both longitudinal (before the pandemic) and cross-sectional (during the pandemic) [32]. Periods of observation ranged from 2 weeks [25] to 9 months [32]. Most studies were carried out in April and/or May 2020 [23,24,25,26,28,29,30,31], while only two studies included March 2020 [27,32]. Authors did not report any information about study duration for one article [33]. 

The number of respondents ranged from 15 [32] to 12,596 [30]. Females represented the majority of respondents in 7 studies [23,25,26,29,30,31,33], while in two studies, male were more represented [24,27]. One study did not report any data regarding this information [32].

The majority of the papers included only ICU or ED staff [23,24,25,27,29,31,32,33], while three studies included workers employed in several medical departments, among which were ED/ICU [26,28,30]. Most of the selected studies included a mixed sample of HCW [23,25,26,28,31,32,33], while four studies included a single HCW, namely physicians [24,27,29] or nurses [30].

Burnout scores were reported as prevalence or mean value. Different kinds of scales were used to assess the level of burnout: the Maslach Burnout Inventory (MBI) was employed in five articles [24,27,29,30,33], the professional quality of life Scale (ProQoL) in two articles [26,28], the Stanford Professional Fulfillment Index (SPFI) and the Well-Being Index (WBI) in one article [32], and the Copenhagen Burnout Inventory (CBI) in one article [31]. For two studies, authors did not declare any validated scale [23,25].

Regarding samples including different professionals, comparable findings were reported by Wahlster [23], Sharma [25], and Chor [31], with a prevalence of burnout ranging between 49.3% [31] and 58% [25]. Similar results were found by studies focused only on physicians [24,29,32], with a prevalence ranging from 51.8% [24] to 57% [32]. However, despite not providing a comprehensive prevalence of burnout, other authors reported lower percentages of highly burned-out professionals [27,30,33]. Indeed, high levels of emotional exhaustion ranged from 3.1% [33] to 24.7% [30], high levels of depersonalization from 12.5% [33] to 21.1% [30], and high levels of lacking personal accomplishments from 1.1% [30] to 25% [33]. In particular, de Wit and colleagues revealed no significant time trend in symptoms during their longitudinal study from March to May 2020 [27].

Considering mean values, most studies reported an intermediate grade of mean values (according to the tools they used) [26,29,30,31], both taking into account more professional positions [26,31] and specific roles, e.g., exclusively anesthetists [29] or exclusively nurses [30]. Conversely, Buselli et al. found low mean values of burnout among ICU staff [28].

Studies that considered different professional positions reported nurses might be more burned-out. Indeed, Chor et al. showed nurses had higher mean scores of burnout compared with physicians [31], and Sharma and colleagues observed that nurses had the highest prevalence of burnout (64%), followed by advanced practice providers and respiratory therapists (respectively, 56% and 55%), physicians (49%) and physicians-in-training (48%) [25]. Last, Wahlster and colleagues reported that nurses had an adjusted relative risk of burnout of 1.31 (95% CI, 1.13–1.53) [23].

**Table 2:** Systematic review on burnout among professionals working in intensive care units and emergency departments: selected studies.

1st Author	Country	Study Design	Setting	Participants	Length of Study	Burnout Evaluation Tool	Prevalence of Burnout	Mean Score for Burnout (SD) [Range]
Sharma M et al. [25]	USA	CS	ICU	Participants: 1651 Female Gender: 74% Mean age: NA Professional title: Physician 25% Nurse 47% Advanced Practice Provider 11% Respiratory therapist 17%	04/23–05/07/20	NA	All participants: 58% Physicians: 49% Physicians-in-training: 48% Nurses: 64% Advanced practice provider: 56% Respiratory therapist: 55%	_
Ruiz-Fernàndez MD et al. [26]	Spain	CS	Primary care centers and other services, including ED/ICU and a COVID-19-specific unit	Participants All participants: 506 ICU/ED/COVID units: 171 (33.7%) Female gender: All participants 76.7% Mean age [range]: All participants 46.7 [23,24,25,26,27,28,29,30,31,32,33,34,35,36,37,38,39,40,41,42,43,44,45,46,47,48,49,50,51,52,53,54,55,56,57] Professional title: All participants: Physician 21.3% Nurse 78.7%	03/30–04/16/20	ProQoL Scale	_	All participants: 24.7 (5.9) ICU: 25.1 (5.4) Emergency department: 24.6 (5.9) Specific COVID-19 unit: 28.9 (7.2)
de Wit K et al. [27]	Canada	MM	ED	Participants: 468 Female gender: 49% Median age [IQR]: 41 [35,36,37,38,39,40,41,42,43,44,45,46,47,48,49,50] Professional title: Physician 100%	03/09–05/17/20	MBI	High emotional exhaustion: Week 4: 18% Week 6: 17% Week 8:14% Week 10: 16% *p* = 0.632 High depersonalization: Week 4: 15% Week 6: 13% Week 8: 10% Week 10: 13% *p* = 0.155 No time trend in burnout levels found	_
Buselli R et al. [28]	Italy	CS	Several departments, including ICU	Participants: All participants 265 ICU 78 (29.4%) Gender female: All participants 68.9% Mean age (SD) [range]: All participants: 40.4 ± (11.2), [19,20,21,22,23,24,25,26,27,28,29,30,31,32,33,34,35,36,37,38,39,40,41,42,43,44,45,46,47,48,49,50,51,52,53] Professional title: All participants: Physician 32.1% Nurse 50.2% Healthcare assistants 17.7%	04/01–05/01/20	ProQoL Scale	_	All participants: 19.8 (5.0) [7,8,9,10,11,12,13,14,15,16,17,18,19,20,21,22,23,24,25,26,27,28,29,30,31,32,33,34,35,36,37,38,39,40,41,42,43,44,45,46,47,48,49,50,51,52,53,54] ICU staff: 19.9 (5.0) (vs. non ICU staff: *p* = 0.586)
Tsan SEH et al. [29]	Malaysia	CS	Anesthesia and ICU	Participants: 85 Female Gender: 63.5% Median age [range]: 31 [27,28,29,30,31,32,33,34,35,36,37,38,39,40,41,42,43,44,45,46,47,48,49,50,51,52,53,54,55,56,57,58] Professional title: Anesthetist 100%	May 2020	MBI	Overall: 55.3% Burnout indices Emotional exhaustion Low 34.1%; Intermediate 34.1%; High 31.8% Depersonalization Low 21.2%; Intermediate 31.8%; High 47.1% Personal accomplishment Low 63.5%; Intermediate 27.1%; High 9.4%	Burnout indices: Emotional exhaustion: 21.35 (9.9), Depersonalization: 8.74 (4.9) Personal accomplishment: 29.2 (7.4)
Azoulay E et al. [24]	Europe, South America, North America, Asia, India, Australia–New Zealand, Africa	CS	ICU	Participants: 1001 MBI respondent: 846 (84.5%) Female gender: 34.2% Median age [IQR]: 45 [39,40,41,42,43,44,45,46,47,48,49,50,51,52,53] Professional title: Anesthetists 100%	04/30–05/25/2020	MBI	Data regarding 846 respondents: Overall burnout: Low: 25.3% Intermediate: 23% High: 51.8% Burnout indices: Emotional exhaustion Low 47,1; Intermediate 29.9%; High: 23% Depersonalization Low 42.7%; Intermediate34.3%; High 23% Symptoms of personal accomplishment Low 33.4%; Intermediate 35.2%; High 31,4% Prevalence of severe BO across region, range Australia–New Zealand, India, Middle Europe, Scandinavia: 20–40% East Europe, North America, Asia, South America, UK, South Europe, the Middle East: 50–70%	_
Chen R et al. [30]	China	CS	Several departments, including CCU	Participants: All participants: 12.596 Critical care units 3577 (28.4%) Intensive care 660 (5.2%) Female gender: All participants 95.6% Mean age (SD): All participants 33.1 (7.5) Professional title: Nurse 100%	April 2020	MBI	Burnout indices: Emotional exhaustion low 47.8% moderate 27.5% high 24.7% Depersonalization low 54.0% moderate 24.8% high 21.1% Lack of personal accomplishment low 96.9% moderate 2.1% high 1.1%	Emotional exhaustion 20.1 (10.3) Depersonalization 5.9 (4.9) Lack of personal accomplishment: 19 (8.3)
Chor WPD et al. [31]	Singapore	CS	ED, UCC	Participants: 337 Female gender: 67.7% Median age: NA Professional title: Physician 37.7% Nurse 62.3%	May 2020	CBI	Moderate to severe burnout 49.3%	49.2 (18.6) Nurses 51.3 (19.6) Physicians 45.7 (16.2) (*p* = 0.005)
Gomez S et al. [32]	USA	MM	ICU	Participants: 21 Female gender: NA Mean age: NA Professional title: Physicians 71%	March–May 2020	SPFI & WBI	57%	_
Cao J et al. [33]	China	CS	Fever clinic	Participants: 37 Female gender: 78.4% Mean age (SD): 32.8 (9.6) Professional title: Physician 43.2% Nurse 51.3% Clinical technicians 5.4%	-	MBI	Data regarding 32 responders Burnout indices: Emotional Exhaustion 3.1% Depersonalization 12.5% Personal Accomplishment 25%	_
Wahlster S et al. [23]	World-wide (77 countries included)	CS	ICU	Participants: 2700 Female gender: 65% Mean age: NA Professional title: Physician 41% Nurse 40% Advanced practice provider: 8% Respiratory therapist: 11%	04/23–05/7/2020	NA	Overall burnout: 52% East Asia and Pacific 30% Europe and Central Asia 48% Latin America and the Caribbean 42% Middle East and North Africa 44% North America 57% South Asia 33% Sub-Saharan Africa 33%	_

Abbreviations: Copenhagen Burnout Inventory (CBI), critical care unit (CCC), cross-sectional study (CS), emergency departments (ED), intensive care unit (ICU), longitudinal study (LS), Maslach Burnout Inventory (MBI), mixed-methods study (MM), not available (NA), professional quality of life (ProQoL), Stanford Professional Fulfillment Index (SPFI) & Well-Being Index (WBI) urgent care center (UCC).

Furthermore, some studies compared burnout among HCWs of different departments. Chen and colleagues reported a significant higher prevalence of nurses with high emotional exhaustion and high depersonalization in critical care units (24.7% and 21.1%) compared with non-critical care units (20.2% and 16.9%) (*p* < 0.001), while they found no significant difference for lack of personal accomplishment (*p* = 0.367). Indeed, working in an ICU was a predictor of emotional exhaustion (OR: 1.23, 95% CI 1.12–1.33; *p* < 0.001) and depersonalization (OR: 1.15, 95% CI 1.06–1.25; *p* = 0.001) [30]. Additionally, Ruiz-Fernandez and colleagues found a significant difference (*p* = 0.028) between mean levels of burnout of staff in ICU (mean = 25.1, SD = 5.4), ED (mean = 24.6; SD = 5.9), a COVID-19-specific unit (mean = 28.9, SD = 7.2), primary care center (mean = 24.5, SD = 6.1), regular hospital care (mean = 24.2; SD = 5.6), health and social care center (mean = 21.8, SD = 5.3) [26]. Instead, Buselli et al. revealed no difference in burnout (*p* = 0.586) between ICU staff (mean = 19.9, SD = 5) and non-ICU staff (mean = 19.7, SD = 4.8) [28].

Regarding cross-country studies, some differences were highlighted. According to Wahlster and colleagues, HCWs working in North America reached the highest prevalence of burnout (57%), followed by European and Central Asiatic professionals (48%) while workers from East Asia and the Pacific had the lowest prevalence (30%) [23]. Azoulay et al. reported Eastern Europe, North America, Asia, South America, the UK, Southern Europe, and the Middle East were in the 50–70% range for severe burnout, while Australia–New Zealand, India, Middle Europe, and Scandinavia were in the 20–40% range [24]. As shown by the two cross-country surveys, the prevalence of burnout varied greatly across Asian countries [23,24], and, considering the single-country studies included in this review, works conducted in South-East Asia revealed that around 50% of participants had burnout [29,31]. Regarding North America, some single-country researches confirmed the above shown findings concerning the USA [25,32], while Canada reported a lower prevalence [27].

Several variables were identified as associated with burnout among ICU/ED staff (Table 3). Age [24] and female gender [23,24] were associated with a higher prevalence of burnout. Insufficient personal protective equipment access [23,25] and other shortages in resources [23,32], stigma from the community [25], worries about financial situation [25], worry regarding COVID-19 [29], poor communication from supervisors [23,25], workload and job demand [23,27,29,32] were associated with a higher risk of burnout. Azoulay et al. find out that clinicians affected by severe burnout were more frequently smoking or taking sleeping pills, whereas alcohol consumption was not influenced [24]. Chor and colleagues observed that staff who were originally working in the ED before the pandemic had a higher rate of burnout compared with those deployed from other departments (90.4% versus 9.6%, *p* = 0.004) [31].

Complete results of critical appraisal are reported in Table 4. The percentage of satisfied criteria ranged from 55% to 80%. None of the studies considered reported any measures taken to categorize and address non-responders and only one record gave information about features of non-responders. Two studies reported justification regarding sample size. On the other hand, 100% of studies satisfied criteria regarding the appropriateness of the study design, the definition of the target population, the selection of sample frame, and coherence between results reported, conclusions, and methods. 

## 4. Discussion

The primary aim of this review was to investigate the prevalence of burnout among ICU/ED HCWs during the COVID-19 pandemic. 

Globally, the prevalence of overall burnout shown by ICU/ED HCWs during the emergency was high, ranging from 49.3% [31] to 58% [25]. These findings are consistent with previous results on burnout amongst this specific category of workers before the pandemic [19,34,35,36]. Indeed, several systematic reviews explored this issue, reporting the lowest values of burnout prevalence from 6% [34] to 25.4% [36] and the highest from 41% [19] to 71.4% [36]. Moreover, considering the different dimensions of burnout, the pre-pandemic prevalence of highly burned-out ICU/ED professionals was remarkable, being approximately around 40% for high emotional exhaustion, high depersonalization, and low personal accomplishment [37,38]. Thus, although it seems clear that a great percentage of ICU/ED staff is currently suffering from burnout, this population has presented a high prevalence of this condition for a long time, and there is not sufficient evidence to understand if such prevalence increased due to the pandemic. Interestingly, in line with this, Magnavita and colleagues concluded that one-third of HCWs (not only ICU/ED) presented burnout during SARS and MERS outbreaks and that such prevalence was similar to the prevalence reported in some categories of HCWs during non-epidemic periods [39]. Moreover, very recently, Amanullah and colleagues explored the burnout issue among general HCWs during the COVID-19 pandemic and, like in the specific population that is the target of the present review, the authors concluded that, despite the COVID-19 having heightened existing challenges that physicians might face, the pandemic was not necessarily associated with increased burnout [40].

Beyond the prevalence of overall burnout, the present review revealed some other issues that should be taken in consideration when planning future research. 

Our results suggest that ICU/ED nurses might be at higher risk for burnout compared with other professional positions. These findings are consistent with other reviews about general HCWs and mental health outcomes during the pandemic. Indeed, Schneider and colleagues reported that wellbeing was at higher risk among nurses than among other HCWs [41] and Danet Danet found more frequent and intense symptoms of several mental conditions among nurses [42]. Such results are not only related to the pandemic context. For instance, in 2016, a systematic review on burnout among ICU professionals highlighted that nurses often work understaffed; they commonly report excessive workload and overtime, and their backbreaking workload is associated with the unpredictable nature of their jobs [34]. Furthermore, during the pandemic, nurses faced a disruption in their everyday activity, as they found themselves in the position of caring closely for patients suddenly deprived of their families, causing a significant emotional burden and a feeling of inadequacy. This situation inevitably led to an increase of psychological distress [43]. As described by Laurent et al. [44], when dealing with end-of-life decisions, nurses tend to consider themselves as simple executing agents, in order to dissociate themselves from decisions in which they did not participate. In this scenario, patients become simply objects of treatment, and nurses feel unable to adequately respond to patient’s needs. Likely, during the pandemic, this feeling was increased, due to the overwhelming number of patients and deaths, leading to job dissatisfaction and burnout.

Moreover, although our results revealed a certain geographical heterogeneity, it is worth noting that the prevalence of overall burnout was always higher than 20%, thus highlighting the presence of a substantial problem across the globe. In addition, it should be noted that the lowest value of overall burnout was reported in Australia [23], and a partial explanation could be linked to the lower number of COVID-19 cases that occurred there. 

Interestingly, considering single-country research, countries that reported a prevalence of burnout around 50% or higher [25,29,31,32] had very different epidemiological situations during the period of observation of the studies [45], both considering differences between countries and within countries from the beginning to the end of the study. For instance, in Malaysia in May 2020 [29], there were remarkably fewer cases compared with the other countries (daily new confirmed COVID-19 cases per million people: beginning of the study = 1.68; end = 2.53). Indeed, in the other countries with a similar burnout prevalence, the daily new confirmed cases per million were around 80 or more, both considering the beginning or the end of such studies [25,31]. The work by Gomez et al. represented an exception and showed the highest variability within study going from less than 1 case per million at the beginning up to 63.14 cases per million at the end [32]. Moreover, in the above-mentioned studies, the stringency index (SI) (from 0 to 100, where 100 means the strictest implemented measures [45]) was always above 70 [25,29,31], except for the study from the USA that started when the SI was 8.33 [32]. However, it is worth noting that the papers that revealed the lowest prevalence of high burnout [27,30] were conducted in countries where, during the period of observation, the number of daily new cases per million was substantially lower compared with the countries with higher burnout (Canada: beginning of the study = 0.21, end = 31.25 [27]; China: beginning = 0.03, end < 0.01 [30]) and countries with a SI of around 70 or lower. Similarly, the epidemiological situation was different between and within studies that presented intermediate mean values of burnout [26,29,30,31] and, in addition, the Italian study that reported a low mean value of burnout [28] was not carried out in a period with fewer cases per million compared with the studies showing intermediate values. Focusing on cross-country research, the link between epidemiological situation [45] and burnout seemed clearer, although such studies considered very wide geographical areas, thus making comparisons less accurate. For instance, in the period of observation of the work by Azoulay and colleagues, North America, South America and the UK were among the countries with the highest prevalence of burnout and also with the highest number of daily new cases per million, while Australia and New Zealand had both the lowest burnout and the lowest number of cases per million [24]. Similarly, Wahlster et al. revealed the highest prevalence of burnout in North America and the lowest in the East Asia and Pacific region, which had more than 50 daily new cases per million (in the USA more than 80) and less than 10, respectively [23]. 

Unfortunately, such studies did not present enough data to understand if burnout varied across the period of observation and epidemiological situation, except for the Canadian study [27] that showed no significant time trend in symptoms from March to May 2020. In addition, we cannot infer about a change in the burnout prevalence over the course of the pandemic since the included papers were conducted until May 2020. Future studies should focus on the relationship between burnout and the different phases of the pandemic.

Finally, this review found several variables associated with burnout. Some socio-demographic variables such as age and female gender have been often found to be associated with burnout, both before the pandemic among ICU/ED workers [34] and during the pandemic among general HCWs [46]. Moreover, age and gender have been associated with many other mental conditions among general HCWs during the COVID-19 pandemic [41,42,47]. Work environment, communication, and support by supervisors have been demonstrated to play a role in burnout among ICU/ED workers before the pandemic [34,37], as well as in other mental health outcomes during the pandemic [48]. Several studies conducted before the pandemic outlined that one of the most important determinants of burnout is represented by workload and job demand [19,34,37]. In the context of the pandemic, workload and job demand have possibly increased and had an impact on the health of general HCWs, concerning both burnout [39,46,49] and other mental issues [50]. Last, we outlined other variables that are more specific for the COVID-19 and other outbreaks and have been reported in other reviews about general HCWs and mental health, such as shortage in resources (e.g., personal protective equipment) [46], perceived threat of COVID-19 [46], and stigma from the community [48].

It must be noted that the SARS-CoV2 strongly hit all health care workforces that had to face a huge and sudden increase of workload in a scenario characterized by uncertainty, and it is clear that burnout represents only one of the possible mental health consequences. Indeed, several reviews focused on other mental health outcomes among HCWs (not only ICU/ED professionals). For instance, depression, anxiety, and post-traumatic stress disorders (PTSD) result turned out to be remarkably high [47,51,52,53], yet not reaching the half of workers as shown for burnout by many works included the present review. Interestingly, both stress and insomnia reached a prevalence comparable to burnout [47,51], further highlighting the urgent need of intervention to care for the mental health of HCWs. Since some studies that we have selected highlighted that burnout was more frequent or severe among ICU/ED staff compared with workers of other departments [26,30], it should be examined more in depth if other mental health outcomes could be more relevant in our target population.

The present review had several limitations that must be acknowledged. The small number of included papers, methodological discrepancies, and the heterogeneity in reporting approaches across the studies precluded a more precise resume and made it impossible to conduct a meta-analysis. In particular, tools for the assessment of burnout have many limitations that have been thoroughly discussed by Mealer and Moss [54] and that are currently even more relevant in the context of the pandemic in light of the high burden that ICU/ED staff must tackle. Indeed, in studies that focus on burnout, it should be excluded that symptoms result from being a novice or from non-work-related concerns and other major psychological problems should be explored along with burnout. Since there is no a comprehensive instrument that measures expertise and events that occur outside of the workplace and since other major disorders are often overlooked, it is difficult to accurately understand how much the burnout data are plausible. In addition, the ICU setting represents a particular environment where even the most used tools, such as the MBI, might be not appropriate. In fact, these instruments do not consider ICU-related triggers (e.g., stress of multiple monitoring alarms, care of families during traumatic situations) or do not take into account that such triggers may overlap with other mental disorders (e.g., depression, anxiety, PTSD) [54].

Moreover, we excluded papers that provided burnout prevalence in overall HCWs (including the ICU/ED staff) but did not give specific data on the burnout of ICU/ED staff. Thus, we did not cover all existing data on ICU/ED workers. Then, it must be noted that the selected studies referred only to early 2020 (up to May); therefore, we did not have an estimate for burnout prevalence across the different phases of the pandemic. Moreover, as burnout is a chronic process by definition [34], acute and cross-sectional measurements during the first months of the pandemic might not fully describe this phenomenon.

Critical appraisal highlighted crucial issues regarding the sampling frame performed using online questionnaires: this method could be considered suitable due to the extraordinary context but may exclude subjects who do not use mail or similar instruments. Indeed, only four studies had a selection process that was likely to select a representative sample. Features of non-responders were not reported, even if this would have been useful to verify how representative the sample was. Moreover, the sudden work overload could have affected response rate and willingness to participate. Last, a complete description of sample-size justification was rarely reported, and descriptions of statistical methods were often non-exhaustive.

## 5. Conclusions

This review highlighted the substantial presence of burnout symptoms in our target population. As the pandemic is still ongoing, a conclusive evaluation of burnout could not be outlined at the moment. However, the present paper provided an overview of data regarding the first phase of the pandemic, a period where the entire world had to face an unknown threat.

It also outlined important and potentially modifiable factors that contribute to burnout onset in the specified settings, such as access to personal protective equipment, staff members’ communication, and organizational aspects. Further research is needed, in particular comparative studies evaluating interventions in different organizational contexts in order to better understand how to tackle and reduce this heavy psychological burden. Several interventions have been demonstrated to be effective in preventing or reducing burnout levels among HCW, both at an individual level (e.g., educational interventions and mindfulness-based interventions) and at an organizational or structural level (e.g., improving workflow management) [49,55,56,57]. However, Pollock and colleagues highlighted a lack of strong evidence about effective interventions for resilience and mental health of HCW during or after epidemics and pandemics [58], and this issue should be further studied. As reflection for future research, it would be interesting to know how many hospitals have implemented interventions against burnout, study in-depth the characteristics of such support interventions, and understand how many centers were prepared to face the burden of burnout during the pandemic. In addition, our results showed that particular attention should be paid to nurses when planning interventions. Last, it would be advisable to share a consistent definition of burnout and related assessment tools to have a better estimate and a more detailed understanding of this issue.

## Figures and Tables

**Table 1:** PICOS strategy.

PICOS Strategy	
Population	HCWs (e.g., physicians, residents, nurses, administrative, pharmacists, therapists) employed in ICU/ED
Intervention	Working in a critical department (ICU/ED) during the COVID-19 pandemic
Comparison	None
Outcomes	Prevalence of burnout or level of burnout during the COVID-19 pandemic
Studies	Any type

Abbreviations: Emergency department (ED), healthcare workers (HCWs), intensive care unit (ICU).

**Table 3:** Systematic review on burnout among professionals working in intensive care units and emergency departments: selected studies.

1st Author	Risk Factors for Burnout in ICU/ED Healthcare Workers
Sharma M et al. [25]	Adjusted relative risk: aRR [IC 95%] Insufficient access to PPE: 1.43 [1.32–1.55]; *p* < 0.01 Poor communication from supervisors: 1.13 [1.06–1.21]; *p* < 0.01 Worries about financial situation: 1.09 [1.01–1.18]; *p* 0.02 Social stigma from community: 1.32 [1.24–1.41]; *p* < 0.01
de Wit K et al. [27]	Factors associated with emotional exhaustion: Having being tested for COVID-19 [OR = 11.5, 95% CI (3.1–42.5)] Number of shifts worked [(OR = 1.3, 95% CI (1.1–1.5) per additional shift, per week] Factors associated with depersonalization: Having been tested for COVID-19 [(OR 4.3, 95% CI (1.1–17.8)]
Buselli R et al. [28]	Burnout presented a significant positive association with the PHQ-9 scores [b = 0.4 (SE = 0.10), *p* < 0.001] and with the GAD-7 scores [(b = 0.20 (SE = 0.06), *p* = 0.001)]
Tsan SEH et al. [29]	Burnout and depression risk were associated each other (*p* < 0.0001). Burnout is associated with number of calls per week (*p* = 0.038) and worry regarding COVID-19 (*p* = 0.014)
Azoulay E et al. [24]	Age and female gender were also associated with a higher prevalence of severe burnout (45 [37,38,39,40,41,42,43,44,45,46,47,48,49,50,51] vs. 47 years [40,41,42,43,44,45,46,47,48,49,50,51,52,53,54,55], *p* = 0.0001, and 38.2% vs. 30.1%, *p* = 0.02). Clinicians with symptoms of anxiety, depression, or severe burnout were more frequently smoking or taking sleeping pills, whereas alcohol consumption was not affected. The number of COVID-19 patients managed was not associated with the prevalence of the psychological burden. Factors independently associated with symptoms of severe burnout included age (HR 0.98/year [0.97–0.99]) and clinician’s rating about the ethical climate (HR 0.76 [0.69–0.82])
Chor WPD et al. [31]	Staff who were originally working in the ED or UCC before the COVID-19 pandemic also had a higher rate of moderate-to-severe personal burnout as compared to those compared to those deployed from other departments (90.4% versus 9.6%, *p* = 0.004)
Gomez S et al. [32]	Among those with burnout, the strongest driver of burnout was related to workload and job demands. Conversely, meaning in work, social support and community at work, and culture and values of work community appeared to be protective of developing burnout as sources of well-being (*p* < 0.001).
Wahlster S et al. [23]	Adjusted relative risk: aRR [IC 95%] Being female 1.16 (1.01–1.33) *p* = 0.03 Being a nurse 1.31 (1.13–1.53) *p* = 0.01 Caring for 10 to 50 patients 1.17 (1.04–1.33) *p* = 0.01 Caring > 50 patients 1.28 (1.06–1.53) *p* = 0.01 Poor communication from supervisors 1.30 (1.16–1.46) *p* < 0.001 Limited availability of PAPRs 1.30 (1.09–1.55) *p* < 0.001 Lack of nurses 1.18 (1.05–1.33) *p* = 0.01 Providers in Europe and Central Asia were 14% less likely to report burnout than were providers in North America 0.86 (0.75–1.00) *p* = 0.04.

Abbreviations: adjusted risk ratio (aRR), emergency departments (ED), generalized anxiety disorders 7 (GAD-7), hazard ratio (HR), intensive care unit (ICU), odds ratio (OR), personal protective equipment (PPE), patient health questionnaire 9 (PHQ-9), urgent care center (UCC).

**Table 4:** Quality assessment grid.

AXIS Items	Sharma M [25]	Ruiz-Fernàndez MD [26]	de Wit K [27]	Tsan SHE [29]	Azoulay E [24]	Chor WPD [31]	Gomez S [32]	Buselli R [28]	Cao J [33]	Chen R [30]	Wahlster S [23]
1. Were the aims/objectives of the study clear?	1	1	1	1	1	1	1	1	0	1	1
2. Was the study design appropriate for the stated aim(s)?	1	1	1	1	1	1	1	1	1	1	1
3. Was the sample size justified	0	0	0	0	0	0	0	0	1	1	0
4. Was the target/reference population clearly defined? (Is it clear who the research was about?)	1	1	1	1	1	1	1	1	1	1	1
5. Was the sample frame taken from an appropriate population base so that it closely represented the target/reference population under investigation?	1	1	1	1	1	1	1	1	1	1	1
6. Was the selection process likely to select subjects/participants that were representative of the target/reference population under investigation?	0	1	1	1	0	0	1	0	0	0	0
7. Were measures undertaken to address and categorize non-responders?	0	0	0	0	0	0	0	0	0	0	0
8. Were the risk factor and outcome variables measured appropriate to the aims of the study?	1	1	1	1	1	1	1	1	1	1	1
9. Were the risk factor and outcome variables measured correctly using instruments/measurements that had been trialed, piloted or published previously?	0	1	1	1	1	1	1	1	1	1	0
10. Is it clear what was used to determined statistical significance and/or precision estimates? (e.g., *p* values, CIs)?	0	1	0	0	1	0	1	1	0	1	0
11. Were the methods (including statistical methods) sufficiently described to enable them to be repeated?	0	1	1	0	1	0	1	1	0	1	1
12. Were the basic data adequately described?	1	1	1	1	0	1	1	1	0	1	1
13. Does the response rate raise concerns about non-response bias?	0	0	1	0	1	1	1	1	0	0	1
14. If appropriate, was information about non-responders described?	0	0	0	0	1	0	0	0	0	0	0
15. Were the results internally consistent?	1	1	1	1	0	1	1	1	1	1	1
16. Were the results for the analyses described in the methods, presented?	1	1	1	1	1	1	1	1	1	1	1
17. Were the authors’ discussions and conclusions justified by the results?	1	1	1	1	1	1	1	1	1	1	1
18. Were the limitations of the study discussed?	1	0	1	0	1	0	0	1	0	1	1
19. Were there any funding sources or conflicts of interest that may affect the authors’ interpretation of the results?	1	1	1	1	1	1	1	1	1	1	1
20. Was ethical approval or consent of participants attained?	1	1	1	1	1	1	1	1	1	1	1
% satisfied criteria	60%	75%	80%	65%	75%	65%	80%	80%	55%	80%	70%

## Data Availability

Not applicable.

## Supplementary Materials

The following are available online at https://www.mdpi.com/article/10.3390/ijerph18158172/s1. Supplementary Material S1: Search strategy.
