# Supplementary material for: The Burden of Burnout among Healthcare Professionals of Intensive Care Units and Emergency Departments during the COVID-19 Pandemic: A Systematic Review
Source: Int J Environ Res Public Health. 2021 Aug 2;18(15):8172. doi: 10.3390/ijerph18158172 (PMC8346023; doi:10.3390/ijerph18158172)
Supplement: Supplementary file 1 [file ijerph-18-08172-s001.zip › ijerph-1276868-supplementary.pdf]

Supplementary Material S1 – Search strategy

**PubMed**
"Anesthesia"[MeSH Terms] OR "Anesthesia"[Title/Abstract] OR "anaesthesia"[Title/Abstract] OR "Anesthesiology"[MeSH Terms] OR "anaesthesiology"[Title/Abstract] OR "Anesthesiology"[Title/Abstract] OR "Anesthetists"[MeSH Terms] OR "anesthetist*"[Title/Abstract] OR "anaesthetist*"[Title/Abstract] OR "anesthesiologist*"[Title/Abstract] OR "anaesthesiologist*"[Title/Abstract] OR "Critical Care"[MeSH Terms] OR "Critical Care"[Title/Abstract] OR "intensive care"[Title/Abstract] OR "intensivist*"[Title/Abstract] OR "Intensive Care Units"[MeSH Terms] OR "Emergency Medical Services"[MeSH Terms] OR "emergency medical service*"[Title/Abstract] OR "emergenc*"[Title/Abstract] OR "resuscitation"[Title/Abstract] OR "resuscitator*"[Title/Abstract] OR "emergency department*"[Title/Abstract] OR "emergency-room"[Title/Abstract] OR "health care workers"[Title/Abstract] OR "health care worker"[Title/Abstract] OR "health worker*"[Title/Abstract] OR "health care provider*"[Title/Abstract] OR "health care personnel"[Title/Abstract] OR "health care professional*"[Title/Abstract] OR "Health Personnel"[MeSH Terms]
**AND**
"severe-acute-respiratory-syndrome-coronavirus-2"[Supplementary Concept] OR "severe-acute-respiratory-syndrome-coronavirus-2"[Title/Abstract] OR "SARS-Coronavirus-2"[Title/Abstract] OR "sars cov 2"[Title/Abstract] OR "nCoV"[Title/Abstract] OR "novel-coronavirus"[Title/Abstract] OR "novel-corona-virus"[Title/Abstract] OR "new-coronavirus"[Title/Abstract] OR "new-corona-virus"[Title/Abstract] OR "2019nCoV"[Title/Abstract] OR "2019-nCoV"[Title/Abstract] OR "coronavirus-disease-2019"[Title/Abstract] OR "corona-virus-disease-2019"[Title/Abstract] OR "coronavirus-disease-19"[Title/Abstract] OR "corona-virus-disease-19"[Title/Abstract] OR "covid*"[Title/Abstract] OR "Coronavirus"[Title/Abstract] OR "COVID-19"[Supplementary Concept] OR "pandemic s"[Title/Abstract] OR "pandemically"[Title/Abstract] OR "pandemicity"[Title/Abstract] OR "pandemics"[MeSH Terms] OR "pandemics"[Title/Abstract] OR "pandemic"[Title/Abstract]
**AND**
"burnout"[Title/Abstract] OR "burn-out"[Title/Abstract] OR "burnout, psychological"[MeSH Terms] OR "Occupational Stress"[MeSH Terms] OR "Occupational Stress"[Title/Abstract]

**Embase**
'anesthesiologist'/exp OR 'intensive care unit'/exp OR 'intensive care'/exp OR 'emergency health service'/exp OR 'anesthesist*':ti,ab,kw OR 'critical care':ti,ab,kw OR 'intensive care':ti,ab,kw OR 'intensivist*':ti,ab,kw OR 'emergency physician*':ti,ab,kw OR 'resuscitation specialist*':ti,ab,kw OR 'anesthesia':ti,ab,kw OR 'anaesthesia':ti,ab,kw OR 'anaesthesiology':ti,ab,kw OR 'anesthesiology':ti,ab,kw OR 'anaesthetist*':ti,ab,kw OR 'anesthesiologist*':ti,ab,kw OR anaesthesiologist*:ti,ab,kw OR 'emergency health service':ti,ab,kw OR 'emergency doctor*':ti,ab,kw OR 'emergency specialist*':ti,ab,kw OR 'resuscitation physician*':ti,ab,kw OR 'resuscitation doctor*':ti,ab,kw OR 'intensive care unit':ti,ab,kw OR 'resuscitator':ti,ab,kw OR 'health care personnel':ti,ab,kw OR 'health care workers':ti,ab,kw OR 'health care worker':ti,ab,kw OR 'health care worker*':ti,ab,kw OR 'health worker*':ti,ab,kw OR 'health care provider*':ti,ab,kw OR 'health care personnel':ti,ab,kw OR 'health care professional*':ti,ab,kw OR 'health care personnel'/exp OR 'emergency department*':ti,ab,kw OR 'emergency medical service*:ti,ab,kw'
**AND**
('burnout':ti,ab,kw OR 'burn-out':ti,ab,kw OR 'burn out':ti,ab,kw OR 'professional burnout'/exp OR 'burnout'/exp OR 'job stress'/exp OR 'job stress':ti,ab,kw OR 'occupational stress':ti,ab,kw)
**AND**
('severe acute respiratory syndrome coronavirus 2':ti,ab,kw OR 'sars-coronavirus-2':ti,ab,kw OR 'sars cov 2':ti,ab,kw OR 'ncov':ti,ab,kw OR 'novel-coronavirus':ti,ab,kw OR 'novel-corona-virus:ti,ab,kw' OR 'new-coronavirus':ti,ab,kw OR 'new-corona-virus:ti,ab,kw' OR '2019ncov':ti,ab,kw OR 'coronavirus-disease-2019':ti,ab,kw OR 'corona-virus-disease-2019':ti,ab,kw OR 'coronavirus-disease-19:ti,ab,kw' OR 'corona-virus-disease-19':ti,ab,kw OR 'coronavirus':ti,ab,kw OR 'covid-19':ti,ab,kw OR 'coronavirus disease 2019'/exp OR 'severe acute respiratory syndrome coronavirus 2'/exp)<br><br>'coronavirus disease 2019'/exp OR 'severe acute respiratory syndrome coronavirus 2'/exp

**Scopus**
TITLE-ABS-KEY ("anesthesia") OR TITLE-ABS-KEY ("anaesthesia") OR TITLE-ABS-KEY ("anaesthesiology") OR TITLE-ABS-KEY ("anesthesiology") OR TITLE-ABS-KEY (anesthetist*) OR TITLE-ABS-KEY (anaesthetist*) OR TITLE-ABS-KEY (anesthesiologist*) OR TITLE-ABS-KEY (anaesthesiologist*) OR TITLE-ABS-KEY ({critical care}) OR TITLE-ABS-KEY ({intensive care}) OR TITLE-ABS-KEY (icu) OR TITLE-ABS-KEY (intensivist*) OR TITLE-ABS-KEY ({emergency-physician*}) OR TITLE-ABS-KEY ({emergency physician}) OR TITLE-ABS-KEY ({emergency-doctor*}) OR TITLE-ABS-KEY ({emergency doctor*}) OR TITLE-ABS-KEY ({emergency-specialist*}) OR TITLE-ABS-KEY ({emergency specialist*}) OR TITLE-ABS-KEY ({resuscitation specialist*}) OR TITLE-ABS-KEY ({resuscitation-specialist*}) OR TITLE-ABS-KEY (resuscitator*) OR TITLE-ABS-KEY ({health care workers}) OR TITLE-ABS-KEY ({health care personnel}) OR TITLE-ABS-KEY ({emergency department*}) OR TITLE-ABS-KEY ({health worker*}) OR TITLE-ABS-KEY ({health care worker*}) OR TITLE-ABS-KEY ({health provider*}) OR TITLE-ABS-KEY ({health care provider*}) OR TITLE-ABS-KEY ({health personnel}) OR TITLE-ABS-KEY ({health care professional*}) OR TITLE-ABS-KEY ({health care personnel}) OR TITLE-ABS-KEY ({emergency medical service*})
**AND**
(TITLE-ABS-KEY ({professional burnout}) OR TITLE-ABS-KEY ({burnout}) OR TITLE-ABS-KEY ({burn out}) OR TITLE-ABS-KEY ({burn-out}) OR TITLE-ABS-KEY ({job stress}) OR TITLE-ABS-KEY ({occupational stress}))
**AND**
((TITLE-ABS-KEY ({severe acute respiratory syndrome coronavirus 2}) OR TITLE-ABS-KEY ({sars coronavirus 2}) OR TITLE-ABS-KEY ({sars-cov2}) OR TITLE-ABS-KEY ({ncov 2019 disease}) OR TITLE-ABS-KEY ({novel coronavirus}) OR TITLE-ABS-KEY ({novel-coronavirus}) OR TITLE-ABS-KEY ({new coronavirus}) OR TITLE-ABS-KEY ({new-coronavirus}) OR TITLE-ABS-KEY ({2019ncov}) OR TITLE-ABS-KEY ({coronavirus disease 2019}) OR TITLE-ABS-KEY ({sars cov 2}) OR TITLE-ABS-KEY ({coronavirus-disease 2019})))

**PsycINFO**
TI "anesthesiology" OR TI "anaesthesiology" OR TI “anesthesia” OR TI “anaesthesia” OR TI anesthetist* OR TI anaesthetist* OR TI anesthesiologist* OR TI anaesthesiologist* OR TI critical care or TI intensive care or TI icu OR TI "emergency services" OR TI "emergency-service*" OR TI "emergency-physician*" OR TI "emergency-doctor*" OR TI "emergency-specialist*" OR TI "resuscitation-physician*" OR TI "resuscitation-doctor*" OR TI "resuscitation-specialist*" OR TI resuscitator* TI "health care workers" OR DE anesthesiology OR DE anesthesiologist OR DE emergency department OR DE emergency room OR DE intensive care unit OR DE icu OR DE critical care OR DE critical care unit OR DE health care workers OR DE medical workers OR DE health care professionals OR AB anesthesiology OR AB "anaesthesiology" OR AB “anesthesia” OR AB “anaesthesia” OR AB anesthetist* OR AB anaesthetist* OR AB anesthesiologist* OR AB anaesthesiologist* OR AB critical care or AB intensive care or AB icu OR AB Emergency Services OR AB "emergency-service*" OR AB emergency-physician* OR AB "emergency-doctor*" OR AB "emergency-specialist*" OR AB "resuscitation-physician*" OR AB "resuscitation-doctor*" OR AB "resuscitation-specialist*" OR AB "resuscitator*" OR AB "health care worker*" OR TI "health care worker*" OR AB "health care worker*" OR TI "health worker*" OR AB "health worker*" OR AB "health care provider*" OR TI "health care provider*" OR TI "health care personnel" OR AB "health care personnel" OR TI "health care professional*" OR AB "health care professional*" OR TI "health professional*" OR AB "health professional*" OR TI "health care personnel" OR AB "health care personnel" OR DE "health care worker"
**AND**
DE "burnout" OR DE "Occupational Stress" OR TI “occupational stress” OR TI "burnout" OR TI "burn-out" OR TI "burn out" OR TI "professional burnout" OR AB “occupational stress” OR AB "burnout" OR AB "burn-out" OR AB "burn out" OR AB "professional burnout"
**AND**
DE "Coronavirus" OR TI “coronavirus” OR AB “coronavirus” OR TI "severe-acute-respiratory-syndrome-coronavirus-2" OR AB "severe-acute-respiratory-syndrome-coronavirus-2" OR TI "SARS-Coronavirus-2" OR AB "SARS-Coronavirus-2" OR TI "sars cov 2" OR AB "sars cov 2" OR TI "nCoV" OR AB "nCoV" OR TI "novel-coronavirus" OR AB "novel-coronavirus" OR TI "novel-corona-virus"OR AB "novel-corona-virus" OR TI "new-coronavirus"OR AB "new-coronavirus" OR AB "new-corona-virus" OR TI "new-corona-virus" OR TI "2019nCoV" OR AB "2019nCoV" OR TI "2019-nCoV" OR AB "2019-nCoV" OR TI "coronavirus-disease-2019" OR AB "coronavirus-disease-2019" OR AB "corona-virus-disease-2019" OR TI "corona-virus-disease-2019" OR AB "coronavirus-disease-19" OR TI "coronavirus-disease-19" OR TI "corona-virus-disease-19" OR AB "corona-virus-disease-19" OR TI covid OR AB covid OR TI "COVID-19" OR AB "COVID-19"
